# Possible Molecular Mechanisms of Hypertension Induced by Sleep Apnea Syndrome/Intermittent Hypoxia

**DOI:** 10.3390/life14010157

**Published:** 2024-01-22

**Authors:** Yoshinori Takeda, Fuminori Kimura, Shin Takasawa

**Affiliations:** 1 Department of Biochemistry, Nara Medical University, 840 Shijo-cho, Kashihara 634-8521, Japan; shintksw@naramed-u.ac.jp; 2 Department of Obstetrics and Gynecology, Nara Medical University, 840 Shijo-cho, Kashihara 634-8522, Japan; kimurafu@naramed-u.ac.jp

**Keywords:** sleep apnea syndrome, intermittent hypoxia, hypertension, reactive oxygen species, hypoxia-inducible factors, pro-inflammatory molecules, renin–angiotensin system, catecholamine, microRNA

## Abstract

Intermittent hypoxia (IH) is a central characteristic of sleep apnea syndrome (SAS), and it subjects cells in the body to repetitive apnea, chronic hypoxia, oxygen desaturation, and hypercapnia. Since SAS is linked to various serious cardiovascular complications, especially hypertension, many studies have been conducted to elucidate the mechanism of hypertension induced by SAS/IH. Hypertension in SAS is associated with numerous cardiovascular disorders. As hypertension is the most common complication of SAS, cell and animal models to study SAS/IH have developed and provided lots of hints for elucidating the molecular mechanisms of hypertension induced by IH. However, the detailed mechanisms are obscure and under investigation. This review outlines the molecular mechanisms of hypertension in IH, which include the regulation systems of reactive oxygen species (ROS) that activate the renin–angiotensin system (RAS) and catecholamine biosynthesis in the sympathetic nervous system, resulting in hypertension. And hypoxia-inducible factors (HIFs), Endotheline 1 (ET-1), and inflammatory factors are also mentioned. In addition, we will discuss the influences of SAS/IH in cardiovascular dysfunction and the relationship of microRNA (miRNA)s to regulate the key molecules in each mechanism, which has become more apparent in recent years. These findings provide insight into the pathogenesis of SAS and help in the development of future treatments.

## 1. Introduction

Intermittent hypoxia (IH) is a condition of repeated hypoxia and re-oxygenation that is typical of sleep apnea syndrome (SAS). SAS typically refers to a collection of conditions characterized by regular pauses in breathing or reduced breathing during sleep. Patients with this condition encounter airway blockage, a lack of oxygen, excessive carbon dioxide, and interrupted sleep due to awakening when there is insufficient oxygen. Approximately 1 billion adults aged between 30 and 69 years are projected to be affected by SAS [1]. SAS is linked to several complications and afflicts many patients. Some of those complications include obesity [2,3], type 2 diabetes [4,5,6,7], dyslipidemia [8,9], dementia [10,11], mood disorders [12], and, especially, hypertension [13,14,15].

Hypertension is the most frequent and important complication of SAS [16], with a prevalence ranging from 30% to 60% [17]. Hypertension in SAS is associated with numerous cardiovascular disorders, including coronary artery disease, stroke, arrhythmias, peripheral artery disease, and heart failure [18,19,20,21]. However, the correlation between SAS and cardiovascular disease is poorly understood due to the complexity of patient studies, which are influenced by factors such as the duration of the disease and the presence of other health conditions. Fletcher et al. proposed the concept that subjecting rodents to cyclical hypoxia would accurately replicate the hypoxemia encountered by patients with SAS [22]. The animals in this model experienced an increase in their blood pressure, which was proportional to the increase observed in patients with SAS [23,24]. The IH model replicates the physiological increase in blood pressure observed in SAS by simulating hypoxic conditions. It is known that the IH model adequately represents cardiorespiratory responses in SAS compared to the continuous hypoxia model [25]. Several studies have been undertaken to elucidate the underlying mechanism of hypertension in patients with SAS using various models.

This is a narrative review, and relevant studies were mainly searched for in PubMed using the keywords correlated with “hypertension” and “intermittent hypoxia”. The search was limited to English-language publications. This review article provides the current understanding of the correlation between IH, a typical and significant pathological condition of SAS, and hypertension. Reactive oxygen species (ROS), hypoxia-inducible factors (HIFs), Endotheline 1 (ET-1), inflammatory molecules, the renin–angiotensin system (RAS), catecholamine, cardiovascular factors, and pro-inflammatory/inflammatory cytokines are mentioned as important mechanisms. Some key molecules among them have been derived through in vivo experiments and further verified in vitro. Also, the review includes an overview of our findings from cellular studies and explores the effects of IH on hypertension. By integrating the related in vivo and in vitro results, we discuss the molecular mechanisms underlying the onset of hypertension due to IH.

## 2. Reactive Oxygen Species (ROS)

ROS refer to unstable and reactive forms of oxygen derivatives that are generated as a result of regular metabolic processes [26,27]. ROS generation is a major cellular mechanism underlying the effects of IH. Several studies have described that the ROS levels were increased in patients with SAS [25,28,29]. It has also been documented that nasal continuous positive airway pressure (CPAP) therapy decreases the levels of the markers associated with oxidative stress [28,30]. The nicotinamide adenine dinucleotide phosphate (NADPH) oxidase (NOX) family of enzymes (EC 1.6.3.1) catalyzes the production of superoxide free radicals by transferring one electron to oxygen from NADPH and generating ROS [31].

The xanthine oxidoreductase enzyme system, which consists of xanthine dehydrogenase (XDH) (EC 1.17.1.4) and xanthine oxidase (XO) (EC 1.17.3.2), is a significant contributor to cellular ROS production [32,33]. A study on rat pheochromocytoma PC-12 cell cultures revealed that IH induces the initial production of ROS by directly stimulating XO [34]. XO activation occurs before NOX2 activation, and it is induced by IH. The ROS produced by XO lead to an increase in the cytosolic calcium concentration and the translocation of the cytosolic subunits p47phox (also known as neutrophil cytosolic factor 1) and p67phox (also known as neutrophil cytosolic factor 2) of NOX to the plasma membrane. These subunits then interact with the catalytic subunit gp91phox of NOX2. Allopurinol (1H-Pyrazolo [3,4-d]pyrimidin-4-ol), an XO inhibitor, or genetic silencing of XO using small interfering RNA (siRNA) can prevent all of these effects [35].

The consequences of brief periods of IH exposure, lasting for 10 days, could be reversed within 10 days of re-oxygenation. However, the consequences of prolonged periods of IH exposure, lasting for 30 days, continue to persist even after 30 days of recovery under normal air conditions [36,37,38,39]. Epigenetic mechanisms are associated with gene regulation and long-term physiological changes. DNA methylation is an example of an epigenetic mechanism. DNA methylation is facilitated by enzymes called DNA methyltransferases (Dnmt1 (DNA cytosine-5 methyltransferase 1; EC 2.1.1.37), Dnmt3a (DNA cytosine-5 methyltransferase 3α; EC 2.1.1.37), and Dnmt3b (DNA cytosine-5 methyltransferase 3β; EC 2.1.1.37)) [40,41]. Rats that received prolonged IH exhibited an increased Dnmt enzyme activity and high levels of Dnmt 1, 3a, and 3b proteins, which corresponded to changes in their respective mRNA levels [36,37,38]. Elevated levels of Dnmt activity are linked to excessive DNA methylation of genes that encode antioxidant enzymes [36,37,38]. Additional examinations revealed that rats subjected to prolonged IH displayed hypermethylation of a solitary CpG dinucleotide near the transcription start site of the superoxide dismutase 2 (SOD-2; EC 1.15.1.1) gene [36,37,38]. In contrast, the effect of short-term IH on DNA methylation was negligible, indicating that only long-term IH triggered DNA methylation. Administering decitabine (Deoxycytidine, 5-aza-2′-deoxycytidine), a DNA methyltransferase inhibitor, to rats during prolonged IH exposure prevents DNA hypermethylation, restores the expression of antioxidant enzyme genes, balances ROS levels in the chemo reflex pathway, and halts the development of hypertension [36,37,38]. These studies indicate that the continuous inhibition of antioxidant enzyme genes such as SOD, catalase (EC 1.11.1.6), and glutathione peroxidase (EC 1.11.1.9) through DNA methylation results in a sustained increase in ROS levels. This ultimately leads to chronic hypertension in rats that have been subjected to long-term IH treatment.

## 3. Hypoxia-Inducible Factors (HIFs)

Hypoxia-inducible factors (HIFs) act as primary controllers that ensure the balance of oxygen levels in all cells of the body by managing the supply and demand of oxygen [39,40,42]. The HIF family of transcription factors regulates the activation of various genes in hypoxic conditions. HIF-1 is composed of two subunits, an oxygen-sensitive inducible factor (HIF-1α) and the constitutive HIF-1β (also known as AhR nuclear translocator (ARNT)). The HIF-1α subunits undergo hydroxylation, ubiquitination, and proteasomal degradation in the presence of oxygen, but they accumulate quickly when oxygen is scarce. In the absence of oxygen, hydroxylation is suppressed, leading to the accumulation of HIF-1α. HIF-1α then combines with HIF-1β, attaches to hypoxia response elements, and triggers the activation of numerous target genes through transcription [42,43]. The molecular mechanism of HIF-1α upregulation during IH was investigated by Yuan et al. [44]. The process involves both an increase in the synthesis of HIF-1α through the mammalian target of rapamycin (mTOR) during re-oxygenation and a decrease in the degradation of HIF-1α through hydroxylase during hypoxia. Mice with HIF-1α heterozygosity that were treated with IH showed a significant lack of elevated levels of ROS [45]. In experiments with cell cultures subjected to IH, the increase in ROS production by NOX has been noted to result in the accumulation of HIF-1α [44]. Furthermore, exposure to IH leads to a decrease in the levels of antioxidants, such as SOD-2 (manganese-dependent superoxide dismutase; EC 1.15.1.1), while also increasing the levels of pro-oxidants, such as NOX [44,46]. Nanduri et al. suggested that the activation of NOX transcription induced by IH requires the simultaneous activation of HIF-1 through lysine demethylases known as the Jumonji-C (JmjC) family of lysine demethylases (JmjC-KDMs) (KDM2-8) [47]. The activation of HIF-1α transcription due to IH is dependent on the recruitment of p300 through Ca^2+^/calmodulin-dependent protein kinase (Cam kinase) II (EC 2.1.11.17) [48]. This research demonstrates that exposure to IH leads to an increase in the levels of HIF-1α through the action of ROS, which in turn affects the transcription of genes responsible for both pro- and antioxidant enzymes.

Prabhakar et al. suggested that IH upregulates HIF-1α but downregulates HIF-2α [49]. The α subunit of HIF-2α, which is regulated by O_2_, is commonly known as endothelial Per-Arnt-Sim (PAS) domain protein-1 (EPAS1) and belongs to the HIF family [50]. HIF-2α shares an 80% sequence similarity with HIF-1α and exhibits interaction with HIF-1β [51]. Similar to HIF-1α, a prolonged lack of oxygen causes an increase in HIF-2α levels, which then triggers the activation of vascular endothelial growth factor (VEGF) through transcription [52]. The downregulation of HIF-2α in response to IH is mediated by calcium signaling. In comparison to the increase in HIF-1α, studies have demonstrated that IH decreases the levels of HIF-2α protein by activating calpains, which are Ca^2+^-activated non-lysosomal cysteine proteases (EC 3.4.22.52/EC 3.4.22.53) that facilitate the degradation of HIF [46]. HIF-2α regulates the gene expression of various antioxidants, such as SOD-2 [46]. IH causes the degradation of HIF-2α, which leads to the inhibition of SOD-2 transcription and ultimately results in increased oxidative stress. ALLM (calpain inhibitor II; N-Acetyl-L-leucyl-L-leucyl-L-methioninal), a membrane-permeable strong calpain inhibitor, reverses the degradation of HIF-2α caused by IH, restores the activity of SOD-2, and prevents the increase in ROS [46]. These studies indicate that IH suppresses the activation of HIF-2α, resulting in elevated levels of ROS. These ROS, in combination with low oxygen levels, function together to stimulate the upregulation of HIF-1α and contribute to the development of hypertension caused by IH.

## 4. Endothelin 1 (ET-1)

Endothelin [53] is composed of 21 amino acids and has two disulfide bonds within the molecule. It is produced by processing a precursor consisting of 203 amino acids. In many mammals, there are three peptide isomers encoded by different genes: ET-1, ET-2, and ET-3 (ET-1: Cys-Ser-Cys-Ser-Ser-Leu-Met-Asp-Lys-Glu-Cys-Val-Tyr-Phe-Cys-His-Leu-Asp-Ile-Ile-Trp (Disulfide bonds between Cys^1^-Cys^15^ and Cys^3^-Cys^11^), ET-2: Cys-Ser-Cys-Ser-Ser-Trp-Leu-Asp-Lys-Glu-Cys-Val-Tyr-Phe-Cys-His-Leu-Asp-Ile-Ile-Trp (Disulfide bonds between Cys^1^-Cys^15^ and Cys^3^-Cys^11^), ET-3: Cys-Thr-Cys-Phe-Thr-Tyr-Lys-Asp-Lys-Glu-Cys-Val-Tyr-Tyr-Cys-His-Leu-Asp-Ile-Ile-Trp (Disulfide bonds between Cys^1^-Cys^15^ and Cys^3^-Cys^11^) in human endothelins). Endothelin has a transient vasodilatory effect followed by a sustained vasoconstrictive effect. However, the vasodilatory effect of ET-3 is very weak compared to the other two types. ET-1 functions through two receptors, namely the endothelin A (ETA) receptor and the endothelin B (ETB) receptor. Research using ETA receptor antagonists has indicated that ET-1 stimulates the ETA receptors. Prolonged exposure to low oxygen levels for 14 days lead to the upregulation of both the ETA receptor and preproendothelin, which is the precursor of ET-1. In addition, an increase in chemoreceptor activity corresponds to an increase in ET-1 and ETA expression [54]. After being subjected to IH for 4 days, cats experienced a 10-fold rise in the expression of ET-1. However, the administration of bosentan (4-*tert*-butyl-*N*-[6-(2-hydroxyethoxy)-5-(2-methoxyphenoxy)-2-(pyrimidin-2-yl)pyrimidin-4-yl]benzenesulfonamide), a competitive and specific antagonist of ETA/ETB receptors in the endothelium and vascular smooth muscle, effectively prevented the IH-induced enhancement of both basal and hypoxic chemosensory responses [55]. This study investigated how ROS impairs the function of baroreceptors in rats that were subjected to IH [56]. The expression of ET-1 was upregulated, and ET-1 causes constriction, similar to its effects on blood vessels. The activation of endothelin converting enzyme (ECE; EC 3.4.24.71), which produces biologically active ET-1, is increased by ET-1 via an ROS-dependent mechanism. The use of an ETA receptor antagonist in rats treated with IH improves their baroreceptor responses. In addition, antioxidants prevented the increase in ET-1 levels and ECE activity caused by IH, reversed the decrease in baroreceptor activity, and restored vasoreflex function in rats. Pawar and colleagues demonstrated that the use of manganese tetrakis methyl porphyrin pentachloride (5,10,15,20-Tetrakis(*N*-methyl-4-pyridyl)-porphyrin-Mn(III) pentachloride), which acts as a scavenger for free radicals, can effectively prevent the IH-induced elevation of ROS, the basal release of ET-1, and the upregulation of ETA mRNA [57]. These results indicate that the increased levels of ET-1 and the upregulation of ETA receptors, which are mediated by ROS, play a role in the heightened sensitivity to hypoxia after exposure to IH.

HIF-1α may play a role in the development of hypertension caused by IH by increasing the expression of ET-1. The pre-pro ET-1 promoter contains hypoxia response elements, and hypoxia increases the transcription of pre-pro ET-1 by recruiting HIF-1α, activator protein 1 (AP-1), GATA-binding factor 2 (GATA-2), CAAT-binding factor (NF-1), and cyclic AMP response element-binding protein (CREB) (p300/CBP/CREB) to the transcriptome [58,59,60]. Furthermore, the increase in ET-1 due to IH relies on the existence of HIF-1 [61]. Consistent with the involvement of HIF-1α and ET-1 in IH, Belaidi et al. showed that HIF-1α and the endothelin system play significant roles in the development of myocardial infarction and hypertension in Wistar Kyoto hypertensive rats exposed to IH [62]. This study shows that the activation of the ET system, facilitated by HIF-1α activity, is the cause of the increased vulnerability to IH and the resulting cardiovascular effects, such as hypertension and ischemic damage.

## 5. Inflammatory Molecules

Inflammation is a natural reaction of the immune system to tissue damage or invasion by pathogens [63]. The typical indicators of inflammation in the clinical setting include heightened blood circulation, the permeability of capillaries, the release of inflammatory substances, and the movement of white blood cells. The activation of molecules by the nuclear factor κ-light-chain-enhancer of activated B cells (NF-κB) leads to the coordination of these processes. This activation triggers the release of inflammatory cytokines such as tumor necrosis factor alpha (TNF-α), interleukin 1β (IL-1β), interleukin 6 (IL-6), interleukin 8 (IL-8), chemokines and adhesion molecules [64]. Oxidative stress has the ability to cause the production and release of pro-inflammatory cytokines by controlling the transcriptional activity of certain transcription factors, such as NF-κB, AP-1, and HIF-1α [65]. NF-κB plays a crucial role in activating HIF-1α transcription and is essential for the buildup of HIF-1α under hypoxic conditions [66,67]. The HIF-1 gene contains a binding site for NF-κB in its proximal promoter region, and NF-κB seems to control the normal levels of HIF-1 gene expression [68]. It seems that the upregulation of HIF-1 transcription during hypoxia is dependent on NF-κB, and vice versa [68,69,70]. Alternatively, hypoxia can directly stimulate the NF-κB factor, leading to the production of pro-inflammatory cytokines through transcription [71]. The occurrence of alternating periods of low oxygen levels and subsequent re-oxygenation in individuals with SAS is linked to a rise in the levels of TNF-α, IL-6, and C-reactive protein in the bloodstream [72,73]. Furthermore, when faced with oxidative stress, HIF-1α triggers the movement of NF-κB to the nucleus, resulting in an increase in the synthesis of pro-inflammatory molecules, such as IL-1β, TNF-α, and ET-1 [74].

According to a study by Lam et al., it was found that after being exposed to IH for a period of 7 days, the levels of TNF-α, IL-1β, and inducible nitric oxide synthase (iNOS, NOS2; EC 1.14.13.39) mRNA increased in rats [75]. The systemic administration of the nonsteroidal anti-inflammatory drug (NSAID) ibuprofen (pyridin-2-ylmethyl(2RS)-2-[4-(2-methylpropyl)phenyl]propanoate) during IH did not decrease the enhanced chemosensory responses to hypoxia. However, it reduced the elevated chemosensory baseline and the elevated levels of pro-inflammatory cytokines [76]. The use of ibuprofen can prevent the development of high blood pressure caused by exposure to IH, as well as the adaptation of the respiratory system in rats. This suggests that ibuprofen may affect other components of the chemoreflex pathway [76]. Ibuprofen also inhibits the increase in the quantity of c-Fos (an AP-1 transcription factor subunit)-positive neurons in the caudal nucleus tractus solitarii (NTSs) of rats exposed to IH [76]. Snyder et al. discovered that IH for a week leads to oxidative stress and elevated levels of pro-inflammatory cytokines in brain regions linked to the initial phases of neurodegeneration, such as the substantia nigra and entorhinal cortex. However, these effects were not observed in the NTS and rostral ventrolateral medulla (RVLM) regions [77]. Oyarce and Iturriaga discovered that the mRNA levels of IL-1β, IL-6, and TNF-α were increased in the NTS of rats with hypertension following 21 days of IH [78]. These results indicate that the presence of pro-inflammatory cytokines in the NTS may play a role in sustaining hypertension. This is supported by the fact that IH leads to an increase in blood pressure within 3–4 days in conscious rats [79].

## 6. Renin–Angiotensin System (RAS)

The RAS has been shown to elevate ROS levels and plays a role in the development of hypertension caused by IH and SAS [80,81,82,83]. Angiotensin II (ANG II), the primary hormone responsible for the actions of RAS, affects both the peripheral and central systems, which can increase sympathetic activity and play a role in the development of hypertension [84]. Patients with SAS experience RAS activation, similar to rats that have been exposed to IH [80,85]. The activation of the carotid body and the exaggerated sympathetic reflexes caused by IH may lead to increased sympathetic nerve activity. This increase could potentially activate the peripheral RAS [86].

Glomus cells, which are contained in the carotid body, possess a pathway that can produce ANG II independently from renin [87,88]. When ANG II is continuously applied, it briefly increases the local firing of carotid afferents. However, when applied at discrete intervals, it leads to a long-lasting increase in baseline sensory nerve activity [89]. The application of serotonin (5-hydroxytryptamine [5-HT]) also produced the same phenomenon [90]. Both serotonin and ANG II work by stimulating the activity of NOX2, which generates ROS. The repeated activation of the glomus cells through ANG II or 5-HT during IH leads to a long-lasting increase in baseline sensory nerve activity and leads to heightened basal and chemoreflex stimulation. This may contribute to the maintenance of elevated mean arterial pressure observed in patients with SAS. Furthermore, ANG II inhibits the baroreflex [91], which may also contribute to the pathogenesis of IH-induced hypertension. The activation of the peripheral RAS could potentially trigger RAS activation in the brain [84]. The angiotensin receptor antagonist, losartan, can block or prevent IH-induced hypertension when administered either peripherally or centrally, indicating that both the peripheral and central RAS are involved in the pathogenesis of this type of hypertension [92,93,94,95]. Similar to ET-1, Lam et al. have demonstrated that ANG II increased the sensitivity of the carotid body to chemical stimuli, specifically within the carotid body itself, rather than due to changes in arterial pressure or blood flow [87]. ANG II increased carotid sinus nerve activity during in vitro carotid body preparation. Angiotensinogen is converted into ANG II by an angiotensin-converting enzyme (ACE; EC 3.4.15.1). Both protein and mRNA levels of angiotensinogen were detected in glomus cells. Similar to ET-1, chronic hypoxia increases the production of Ang II type 1 (AT1) receptors in the carotid body at the transcriptional and post-transcriptional levels [96]. Furthermore, blocking the AT1 receptors prevented the IH-induced increase in ROS production. This suggests that ANG II may enhance the chemoreceptor function. Shimoura et al. proposed that IH leads to a gradual activation of the RAS, which in turn stimulates a certain level of central AT1 receptor signaling. This signaling threshold is believed to have a permissive role in the initiation of a long-lasting increase in baseline sensory nerve activity [97]. Shell et al. suggested that injections of short hairpin RNA (shRNA) against AT1a into the median preoptic nucleus (MnPO) inhibited the IH-associated elevation in mRNA. Injections of shRNA prevented the sustained component of hypertension during normoxia, and reduced the levels of circulating advanced oxidation protein products, which are indicators of oxidative stress. This indicates that AT1 receptors in the MnPO contribute to the sustained increase in blood pressure during IH [98].

**Figure 1:**
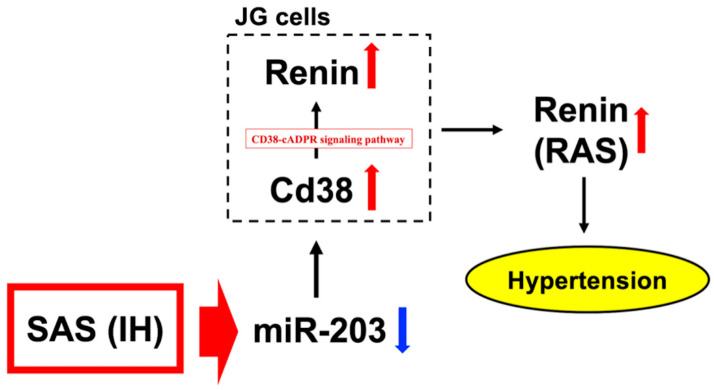
Involvement of RAS in SAS/IH-induced hypertension. The decrease in miR-203 in IH-treated renin-producing JG cells increased gene expression for Renin and Cd38. The upregulation of Cd38 (ADP-ribosyl cyclase) results in the increase in cADPR and Renin expression. It is proposed that the increased expression of Renin and Cd38 in JG cells may contribute to the development of hypertension, while miR-203 may have a significant impact on the modulation of these gene expressions in patients with SAS [112]. The up and down arrows next to the name indicate increase and decrease, respectively.

Renin (angiotensinogenase; EC 3.4.23.15) is considered the main factor that influences the RAS. Renin speeds up the RAS process by catalyzing the conversion of angiotensinogen into angiotensin I [99]. Renin is produced and released in the juxtaglomerular (JG) cells found in the afferent arteriole of the glomerulus in the kidney. Furthermore, the cluster of differentiation 38 (CD38)-cyclic ADP-ribose (cADPR; 1-β-D-ribofuranosyl-adenosine 5′-[trihydrogen diphosphate])-mediated signaling pathway has been proposed to have a function in regulating the synthesis and/or secretion of renin [100]. cADPR acts as a secondary messenger that triggers the release of calcium ions from the endoplasmic reticulum through ryanodine receptors (RyRs) [101,102,103,104,105]. CD38 is a type II glycoprotein that produces cADPR from NAD^+^ (ADP-ribosyl cyclase; EC 3.2.2.6) [106,107,108,109]. The expression of renin in As4.1 cells, a mouse prototype of juxtaglomerular (JG) cells, is influenced by the CD38-cADPR-mediated signaling pathway [110]. In our previous study, we examined the mRNA expression levels of Renin and Cd38 in the renin-producing As4.1 cells and investigated the mechanisms that regulate their expression when treated with IH. The cells were exposed to either normoxia (21% O_2_, 5% CO_2_, and balanced N_2_) or IH (70 cycles of 5 min sustained hypoxia (1% O_2_, 5% CO_2_, and balanced N_2_) and 10 min normoxia) in a custom-designed, computer-controlled incubation chamber attached to an external O_2_-CO_2_-N_2_ computer-driven controller, and we use this setting in our subsequent research. The expression of Renin and Cd38 mRNA is notably elevated in human HEK293 (established from transformed embryonal kidneys due to adenovirus (type 5)) and mouse As4.1 renal JG cells, established from transgenic mice with simian virus (SV)40 T-antigens under the control of a renin promoter, following IH stimulation, suggesting their potential involvement in the CD38-cADPR signaling pathway. To examine the relationship between CD38 and renin expression, we introduced siRNA-targeting Cd38 into As4.1 JG cells and found that the IH-induced increase in renin expression was notably inhibited when small interfering RNA for Cd38 (siCd38) was introduced into As4.1 cells just before IH exposure. Furthermore, we found that the presence of 8-bromo-cyclic ADP-ribose (8-Br-cADPR), a cell-permeable antagonist of cADPR [111], inhibited the IH-induced increase in expression of both Renin and Cd38. The mRNA levels of Ryr(s), an important component of the CD38-cADPR signaling pathway releasing Ca^2+^ from an intracellular pool in response to cADPR, remained unchanged after exposure to IH. These findings suggest that the increase in Renin expression under IH conditions may be due to an increase in Cd38 expression and the subsequent activation of the CD38-cADPR signaling pathway, resulting in the upregulation of cADPR in As4.1 JG cells. We also found that IH did not enhance the promoter activity of either the renin or Cd38, suggesting that the increase in Renin and Cd38 mRNA levels caused by IH is controlled post-transcriptionally. As a post-transcriptional factor, we focused on microRNA (miRNA (miR)). Renin and Cd38 both have a possible target sequence for miR-203, and the IH-treated cells showed a significant decrease in miR-203 levels compared to the normoxia-treated cells. The introduction of the miR-203 mimic prevented the IH-induced increase in Renin and Cd38 mRNAs, and the introduction of the non-specific control RNA for the miR-203 mimic did not prevent this upregulation. These findings suggest that IH causes a decrease in miR-203 expression in cells that produce renin, leading to an increase in Renin and Cd38 mRNA levels and ultimately resulting in hypertension [112] (Figure 1).

## 7. Catecholamine

**Figure 2:**
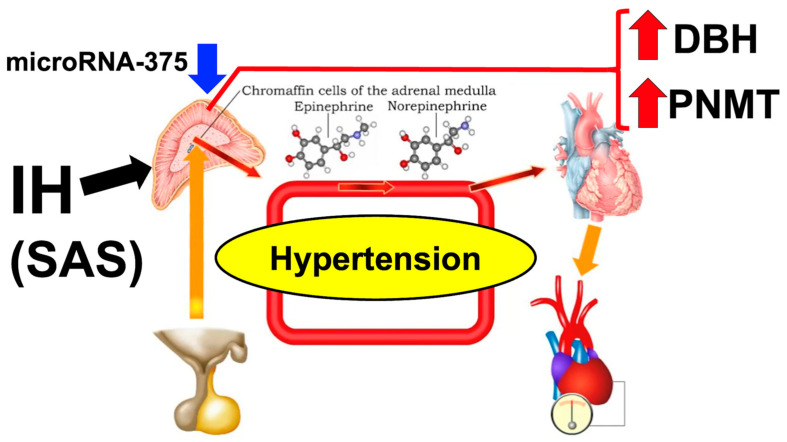
Possible mechanism of IH-induced hypertension in catecholamine-producing cells. The downregulation of miR-375 in IH-treated catecholamine-producing cells increased gene expression for DBH and PNMT. It is proposed that the increased expression of DBH and PNMT in neural cells in the adrenal medulla may contribute to the development of hypertension, while miR-375 have a significant impact on the modulation of these gene expressions in patients with SAS [115].

Catecholamines also play a role in regulating blood pressure [113,114]. We examined the expression of enzymes involved in catecholamine production in both human and mouse neuroblastoma cells. Catecholamine-producing mouse Neuro-2a and human NB-1 neuroblastoma cells were used to prepare the cellular RNA. Cells were exposed to IH for 24 h, which is known to produce catecholamines. The levels of catecholamine biosynthesis enzymes, tyrosine hydroxylase (TH; EC 1.14.16.2), L-3,4-dihydroxyphenylalanine (DOPA) decarboxylase (DDC; EC 4.1.1.48), dopamine β-hydroxylase (DBH; EC 1.14.17.1), and phenylethanolamine N-methyltransferase (PNMT; EC 2.1.1.28) mRNAs were measured using a real-time reverse transcription polymerase chain reaction (RT-PCR). The real-time RT-PCR showed that exposure to IH led to a significant increase in the expression levels of DBH and PNMT mRNA levels in NB-1 and Neuro-2a cells. Immunoblot analyses revealed that IH significantly enhanced the expression of DBH and PNMT in NB-1 cells. The promoter assays using firefly luciferase as a reporter showed that the transcription of the DBH and PNMT genes in response to IH was not responsible for regulating their expressions. In relation to the mechanism involving miRNAs, the levels of miR-375, which was identified using the MicroRNA.org program as targeting both DBH and PNMT mRNAs, were measured using real-time RT-PCR in cells treated with IH. The level of miR-375 was significantly reduced compared to that in cells treated with normoxia. The increase in DBH and PNMT caused by IH was reversed when the miR-375 mimic was introduced to cells, but was not affected by the introduction of the non-specific control RNA for the miR-375 mimic. These findings suggest that IH promotes an increase in DBH and PNMT levels by preventing the degradation of DBH and PNMT mRNAs, which is normally mediated by miR-375 [115]. Hence, it can be concluded that the upregulated expression of DBH and PNMT in the neural cells of the adrenal medulla in patients with SAS may lead to high blood pressure. Additionally, miR-375 may play an important role in controlling the gene expression of DBH and PNMT. IH in patients with SAS and experimental models has been found to cause an increase in catecholamine synthesis, the secretion of catecholamines, and the upregulation of the PNMT gene, as reported in multiple studies [116,117]. Under hypoxic conditions, the secretion of catecholamines from neuronal and adrenal chromaffin cells is increased. This increase in secretion is observed both in laboratory settings (in vitro) and in living organisms (in vivo) and is accompanied by an upregulation of gene expression related to catecholamine production [118,119,120] (Figure 2).

## 8. Cardiovascular Factors for Hypertension in IH

Vascular function assessments are helpful for a better understanding of the pathophysiological associations between vascular dysfunction and cardiac diseases, which could cause hypertension. Kyotani et al. isolated and cultured rat primary vascular smooth muscle cells and exposed them to IH. They found that IH increased vascular smooth muscle cell proliferation by upregulating the epidermal growth factor (EGF) family (epiregulin (EREG), amphiregulin, and neuregulin-1) and their erbB2 receptor (CD340) [121]. They also discovered that IH markedly elevated the expression of IL-6 and EREG in SV40-immortalized human coronary artery smooth muscle cells. Furthermore, the addition of IL-6 to the coronary artery smooth muscle cells induced EREG expression. These findings indicate that IL-6 may have a crucial role in EREG upregulation due to IH and, consequently, SAS-related atherosclerosis [121].

**Figure 3:**
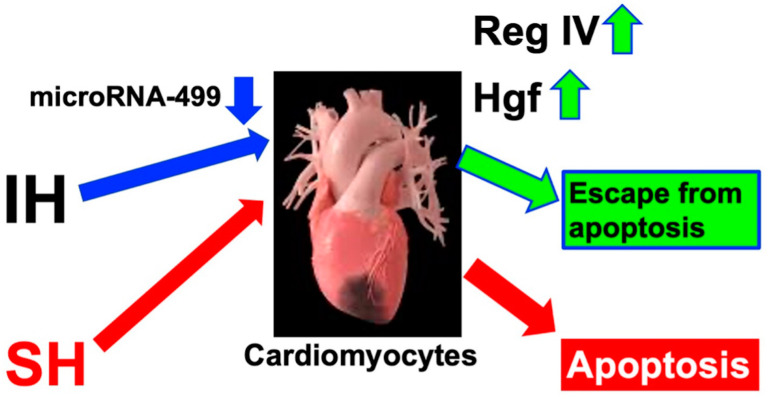
Possible mechanism as to how cardiomyocytes escape from IH-induced apoptosis. The downregulation of miR-499 in IH-treated cardiomyocytes increased gene expression for Reg IV and Hgf. Both proteins of Reg IV and Hgf acted as anti-apoptotic factors in the cardiomyocytes in sustained hypoxia (SH). An increased expression of Reg IV and Hgf due to IH is proposed, and they may act to inhibit the apoptosis of cardiomyocytes, potentially contributing to the maintenance of cardiomyocyte numbers and functions, and miR-499 have a significant impact on the modulation of these gene expressions in patients with SAS [127]. The up and down arrows next to the name indicate increase and decrease, respectively.

Regenerating gene (Reg) family proteins [122] and hepatocyte growth factor (Hgf) work as growth and anti-apoptotic factors in several tissues, such as pancreatic β-cells [123,124,125], the periosteum [126], and intestinal epithelial cells [122]. We found a significant increase in the mRNA levels of regenerating gene IV (Reg IV) and Hgf in rat H9c2 and mouse P19.CL6 cardiomyocytes that occurred due to IH. However, the promoter activities of these genes did not increase. We searched miRs against Reg IV and Hgf mRNAs, revealing that the rat and mouse mRNAs contain a potential target sequence for miR-499. The level of miR-499 in cardiomyocytes treated with IH was notably reduced compared to cells treated with normoxia. The P19.CL6 cells were transfected with an miR-499 mimic and a non-specific control RNA (miR-499 mimic NC). The upregulation of genes induced by IH was canceled by introducing the miR-499, but not by the miR-499 mimic NC. The findings suggest that IH stress leads to the suppression of miR-499 in cardiomyocytes, subsequently elevating the expression of Reg IV and Hgf mRNAs. This molecular response may contribute to the cardioprotective effects observed in patients with SAS [127] (Figure 3).

**Figure 4:**
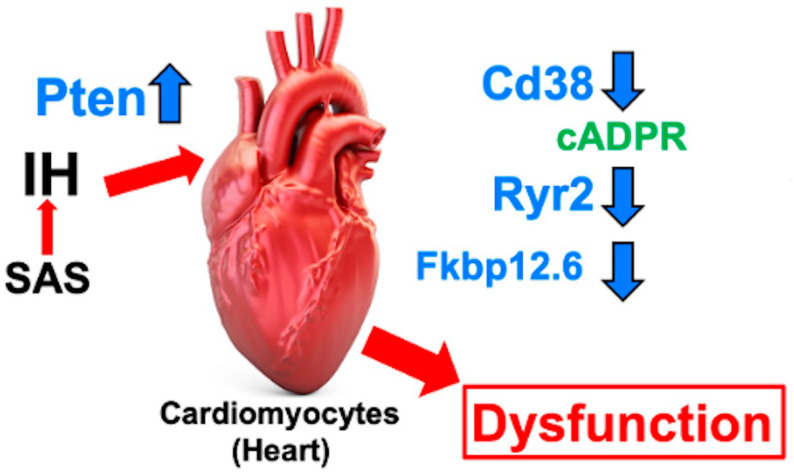
Possible mechanism of cardiomyocyte dysfunction due to IH. The upregulation of Pten in IH-treated cardiomyocytes decreased gene expression for Cd38, Ryr2, and Fkbp12.6. It is proposed that the decreased expression of Cd38, Ryr2, and Fkbp12.6 could downregulate the function of the Cd38–cADPR signaling in cardiomyocytes, potentially contributing to the decreased cardiac functions in patients with SAS [131]. The up and down arrows next to the name indicate increase and decrease, respectively.

The CD38-cADPR signaling system was originally found in glucose-induced insulin secretion from pancreatic β-cells [101,102,109]. Subsequently, it became clear that the CD38-cADPR signaling system is important for cardiac functions [128,129]. We found that the mRNA levels of Cd38, Ryr2 (cardiac-type ryanodine receptor), and FK506-binding protein 12.6 (Fkbp12.6; cADPR receptor) [109], essential components of RyR2 in H9c2 and P19.CL6 cardiomyocytes, were notably reduced due to IH, while the promoter activities of these genes remained unaffected. On the other hand, IH-treated cardiomyocytes exhibited an elevation in the expression of the phosphatase and tensin homolog deleted from chromosome 10 (Pten). Small interfering RNA against Pten (siPten) and a non-specific control RNA were introduced into the H9c2 cells. The reduction in Cd38, Ryr2, and Fkbp12.6 induced by IH was negated by the introduction of the siPten, but not by the control RNA. In addition, we added 3-deaza-cADPR [130], a cell-permeable cADPR agonist, into an H9c2 cell culture medium, followed by subjecting the cells to normoxia or IH for 24 h. After exposure to IH, the mRNA levels of Cd38, Ryr2, and Fkbp12.6 were assessed. It was found that the IH-induced reduction in the mRNA levels of Cd38, Ryr2, and Fkbp12.6 were mitigated by the introduction of 3-deaza-cADPR. This indicates that the IH-induced decrease in these mRNA levels may be attributed to the downregulation of the Cd38–cADPR-mediated signaling pathway. These results suggest that IH stress increases Pten expression in cardiomyocytes, reducing the mRNA levels of Cd38, Ryr2, and Fkbp12.6, leading to impaired cardiomyocyte function in patients with sleep apnea syndrome [131] (Figure 4).

**Figure 5:**
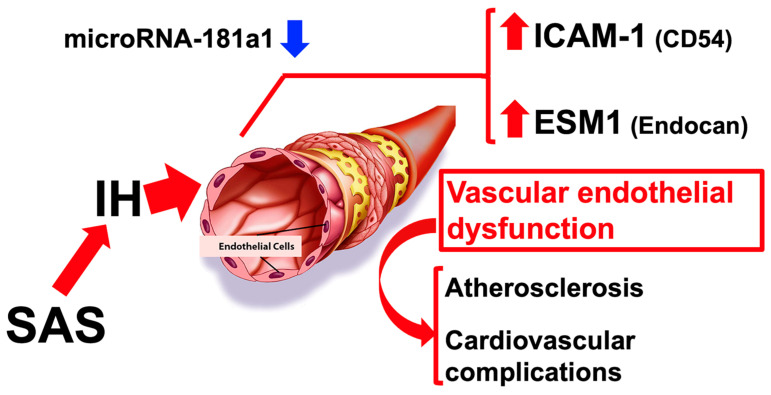
Possible mechanism of vascular endothelial dysfunction induced by IH. The downregulation of miR-181a1 in IH-treated vascular endothelial cells increased gene expression for Esm1 and Icam-1. It is suggested that, in patients with SAS, the increased expression of Esm1 and Icam-1 in vascular endothelial cells may contribute to the development of vascular endothelial dysfunction, while miR-181a1 may have a significant impact on the modulation of these gene expressions [133]. The up and down arrows next to the name indicate increase and decrease, respectively.

Vascular endothelial dysfunction, the earliest predictor of the subsequent development of cardiovascular diseases and hypertension [132], has been observed in patients with SAS. However, the specific mechanism through which IH induces endothelial complications remains unclear. Recently, we exposed vascular endothelial cells (human HUEhT-1 and mouse UV2) to IH, analyzed their gene expression, and found that IH exposure increased the expression of intercellular adhesion molecule 1 (Icam-1/CD54), a cell surface glycoprotein known as an adhesion receptor that directs leukocytes from circulation to sites of inflammation, and endothelial cell-specific molecule 1 (Esm1/Endocan). Icam-1 is present at minimal levels in immune cells, endothelial cells, and epithelial cells, but its expression is recognized to increase in reaction to inflammatory stimuli. The primary focus of research on Icam-1 has been its role in facilitating the trans-endothelial migration of leucocytes. Icam-1 is known to modulate the rolling and adhesion of leucocytes to the vessel wall, as well as to facilitate their passage through the endothelial layer. Esm1/Endocan is a proteoglycan associated with endothelial cells and is increased in response to proangiogenic molecules and pro-inflammatory cytokine stimulation. Esm1 is regarded as a novel biomarker with relevance to both tissue and blood, as it served as an indicator of endothelial activation and dysfunction. We conducted additional investigation into the pathways through which IH increased the mRNA levels of both Icam-1 and Esm1. We also identified a potential post-transcriptional mechanism involving miR-181a1-mediated expression [133] (Figure 5).

## 9. Pro-Inflammatory/Inflammatory Cytokines

**Figure 6:**
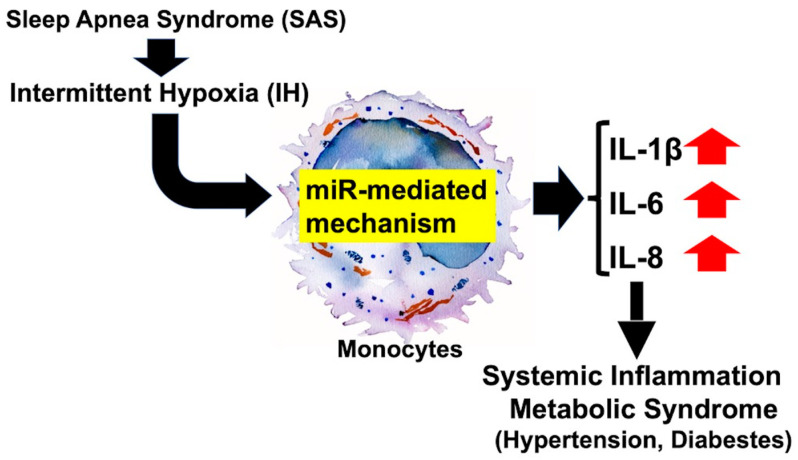
Possible mechanism of systemic inflammation and metabolic syndrome induced by IH. The gene expression of IL-1β, IL-6, and IL-8 were increased in monocytes through miR-mediated mechanism due to IH. Upregulation of IL-1β, IL-6, and IL-8 in monocytes could lead patients to “systemic inflammation”. The up arrows next to the name indicate increases.

Several pro-inflammatory/inflammatory markers, including IL-1β, IL-6, IL-8, IL-17, IL-23, interferon-γ, CXCL10, and TNF-α, were found to be elevated in SAS patients with hypertension [134]. Most recently, we found that IH upregulated IL-1β, IL-6, and IL-8 in human monocytes through an miR-mediated mechanism. IL-1β induced type 2 NOS in pancreatic β-cells that exerted inhibitory and cytotoxic effects on insulin-producing pancreatic β-cells [135], which initiates or worsens diabetes. IL-6 induced EREG in coronary artery smooth muscle cells [121] to induce atherosclerosis. IL-8 was reported to be induced by IH in skeletal muscle cells to induce and worsen insulin resistance [136]. Therefore, the increases in pro-inflammatory and inflammatory cytokines such as IL-1β, IL-6, and IL-8 in monocytes lead to systemic inflammation and worsen metabolic syndrome, including hypertension in SAS patients (Figure 6).

## 10. Conclusions

**Figure 7:**
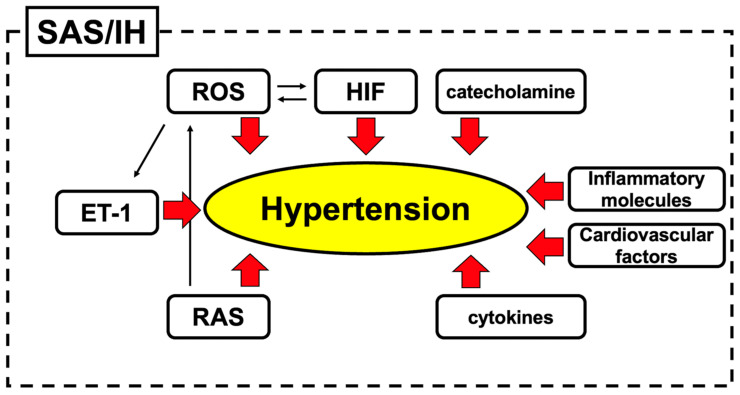
Summary of possible molecular mechanisms of hypertension induced by sleep apnea/intermittent hypoxia.

We have discussed the molecular mechanisms of hypertension induced by sleep apnea/intermittent hypoxia, which are summarized in Figure 7, including their cross-relationships. In this review, we mainly mentioned the molecular mechanisms revealed in vitro. Although in vitro experiments cannot wholly reproduce in vivo phenomena, they have uncovered many key molecules for hypertension in IH. Hypertension associated with SAS causes various health hazards. Therefore, it is crucial to elucidate its onset mechanisms and explore treatments. Several studies have elucidated the mechanisms of hypertension caused by IH. In addition to what has been revealed so far, there is still room to explore the epigenetic regulation mechanisms, including microRNAs, caused by IH, and future research is expected. The identified key molecules can be used as therapeutic targets for future advances in drug discovery. A further elucidation of the pathogenesis of hypertension in patients with SAS will lead to the discovery and development of novel therapeutic strategies.

## Data Availability

Not applicable.
